# *M. tuberculosis* Transcription Machinery: A Review on the Mycobacterial RNA Polymerase and Drug Discovery Efforts

**DOI:** 10.3390/life12111774

**Published:** 2022-11-03

**Authors:** Filia Stephanie, Usman Sumo Friend Tambunan, Teruna J. Siahaan

**Affiliations:** 1 Department of Chemistry, Faculty of Mathematics and Natural Sciences, Universitas Indonesia, Depok 16424, Indonesia; 2 Department of Pharmaceutical Chemistry, School of Pharmacy, The University of Kansas, Lawrence, KS 66045, USA

**Keywords:** *M. tuberculosis*, DNA-dependent RNA polymerase, transcription inhibition

## Abstract

*Mycobacterium tuberculosis* (MTB) is the main source of tuberculosis (TB), one of the oldest known diseases in the human population. Despite the drug discovery efforts of past decades, TB is still one of the leading causes of mortality and claimed more than 1.5 million lives worldwide in 2020. Due to the emergence of drug-resistant strains and patient non-compliance during treatments, there is a pressing need to find alternative therapeutic agents for TB. One of the important areas for developing new treatments is in the inhibition of the transcription step of gene expression; it is the first step to synthesize a copy of the genetic material in the form of mRNA. This further translates to functional protein synthesis, which is crucial for the bacteria living processes. MTB contains a bacterial DNA-dependent RNA polymerase (RNAP), which is the key enzyme for the transcription process. MTB RNAP has been targeted for designing and developing antitubercular agents because gene transcription is essential for the mycobacteria survival. Initiation, elongation, and termination are the three important sequential steps in the transcription process. Each step is complex and highly regulated, involving multiple transcription factors. This review is focused on the MTB transcription machinery, especially in the nature of MTB RNAP as the main enzyme that is regulated by transcription factors. The mechanism and conformational dynamics that occur during transcription are discussed and summarized. Finally, the current progress on MTB transcription inhibition and possible drug target in mycobacterial RNAP are also described to provide insight for future antitubercular drug design and development.

## 1. Introduction

Tuberculosis (TB) is a communicable disease that infects patients through inhalation of the expelled droplets containing the *Mycobacterium tuberculosis* (MTB) bacilli from an active TB patient. According to a previous hypothesis, the *Mycobacterium* genus was supposed to have originated more than 150 million years ago [1]. Despite being an ancient disease, TB still ranks as the top disease-causing high mortalities from a single infectious agent. More than 10 million people acquired this disease in 2019, with the estimated fatality reaching 1.4 million in 2020 [2]. In past years, some drugs such as bedaquiline, pretomanid, and their combination use with linezolid have been utilized for TB patients; unfortunately, due to the emergence of drug-resistant strains, TB has caused a global crisis [3,4,5]. Beyond the global pandemic and drug-susceptibility problem, TB treatment compliance also plays a significant role in reducing TB cases [6]. TB treatment is known to involve a combination of antitubercular drugs for a long period of time (i.e., 6 to 24 months); however, most of these drugs are known to be associated with several adverse effects due to their toxicity levels [7,8]. Treatment interruption could be implemented due to the presence of comorbidity, such as TB/HIV, in patients. In addition, non-compliance is commonly found in patients undergoing long-term TB therapy [9,10]. Due to these problems, the effort to discover a novel antitubercular drug with a shorter treatment regimen is imperative [11].

Knowing how the mycobacterial cell works is a prominent point of departure for drug discovery attempts for designing TB treatments. Every cell function of an organism is dictated by proteins and protein synthesis in a living system in a highly regulated manner [12,13]. In the case of TB, despite the problem with cell membrane permeability, inhibiting protein synthesis has been shown as an attractive way to discover and develop antibiotics [14]. Transcription is one of the crucial steps in protein synthesis, where mRNA is synthesized from the template DNA through initiation, chain elongation, and termination processes [15]. In MTB, the main key enzyme for the bacterial transcription process is DNA-directed RNA polymerase (RNAP) enzyme [16]. RNAP activity and specificity are regulated by the transcription factors that have particular structural motifs according to the sequence on which they bind [17,18]. Rifampicin (RIF) inhibits the activity of the RNAP subunit β (RpoB) of MTB as the important line of defence for TB. RIF has a higher sensitivity towards MTB RpoB compared to *E. coli* RpoB [19]. RIF possesses an excellent bactericidal activity towards MTB with a low MIC value [20]. The activity of RIF is lowered due to the increase in multi-drug resistance/RIF resistance TB (MDR/RR-TB) strains from mutations in the RIF binding site of RpoB, which results in structural incompatibility and treatment failure [21,22].

During the transcription process, RNAP undergoes a sequence of events to activate its catalytic-competent form and begin the RNA synthesis. This process involves engagement with the DNA promoter, unwinding the double-stranded DNA, loading the template into the active site, NTP substrate uptake, and the nucleotidyl transfer to synthesize the phosphodiester bond in the nascent RNA. As targeting the transcription process is still one of the attractive strategies to develop a new inhibitor to combat TB, this review aims to discuss the nature of MTB RNAP as well as the mechanism of the bacterial transcription process and current drugs to target MTB RNAP. The current progress of drug discovery and development related to bacterial transcription inhibition is also summarized to provide an insight into the advancement on antitubercular drugs.

## 2. MTB Transcription Machinery

### 2.1. Mycobacterial RNAP

RNAPs are found as highly conserved molecular machinery, which is important for the transcription process as a part of the gene expression system in living organisms. In both eukaryotes and prokaryotes, RNAPs work as multi-unit enzymes. Eukaryotic cells undergo a more complex transcription process, as they possess three different RNAPs (i.e., RNAP I, II, and III) with a distinct number of subunits, in which each RNAP is assigned for a specific transcription material [23]. In contrast, archaeal RNAP only uses one type of RNAP for the transcription; it consists of 13 subunits forming a horseshoe-shaped architecture. This form is structurally conserved and was found in eukaryotic RNAP II due to evolutionary change [24]. Similar to archaeal RNAP, bacteria also utilize one type of bacterial RNAP that consists of five subunits, making it the most straightforward among all RNAPs [25]. Although different in sizes, RNAPs for eukaryotic, archaeal, and bacterial cells are related to each other with the same mechanism. All RNAPs possess an overall claw-like shape and contain two magnesium (II) ions in the catalytic site coordinated through a conserved aspartic acid triad [26,27].

**Figure 1:**
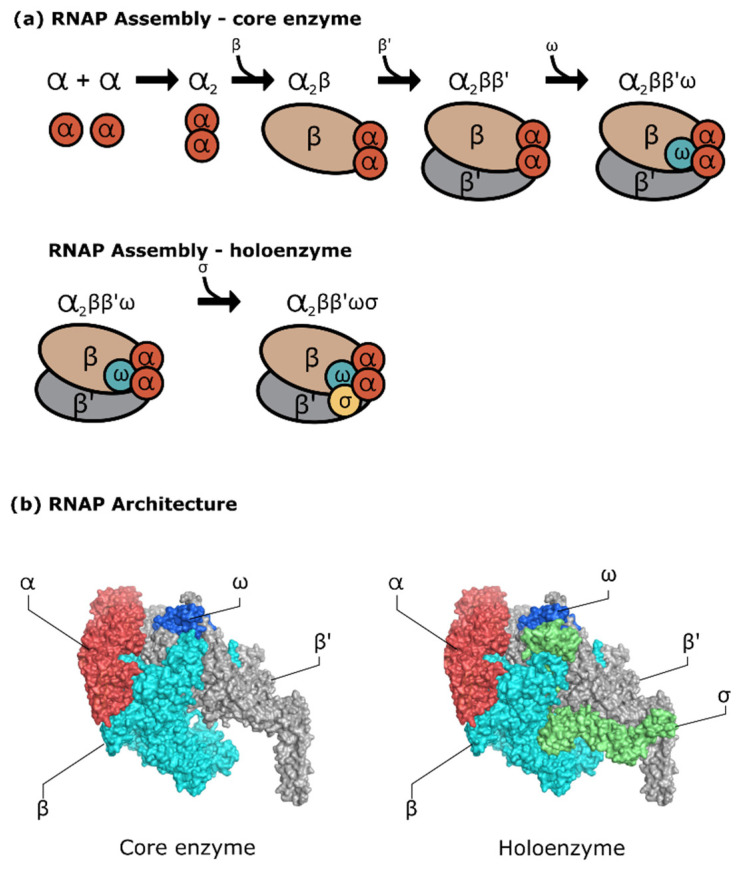
(**a**) RNAP core and holoenzyme assembly; (**b**) RNAP core and holoenzyme architecture (PDB ID: 6C05 [30]).

Despite sharing the same lineage and structural similarities, mechanistic studies found that the MTB RNAP exhibited several differences compared to two of the most studied RNAP—*E. coli* RNAP and *Thermus* RNAP—ranging from the regulatory system, kinetics during initiation, and the presence of crucial transcription factors such as CarD and RbpA protein [28]. The ~400 kDa mycobacterial RNAP core enzyme, as any bacterial RNAP, has five subunits with two identical α subunits, β, β′, and ω subunits. As visualized in Figure 1, the five subunits are associated to form a claw-like structure, with the bigger subunits forming the clamp that facilitates the DNA binding. Another characteristic of this enzyme is in the existence of the primary and secondary channels accessing the active site, where the first accommodates access for the DNA–RNA hybrid and downstream DNA, and the latter accommodates the NTP substrate [29]. Movement of the clamp to ‘open’ and ‘close’ positions is facilitated by the ‘flexible’ domain at the clamp base (switch region). The clamp is open before the promoter binding and remains closed after the promoter interaction, initiation, and elongation step.

The core enzyme assembly (Figure 1a) starts with the two copies of the α subunit—that is, ~36.5 kDa–containing 329 amino acid residues. This α subunit contains the N- and C-terminal domains with residues 20–235 and 236–329, respectively. Both domains are conjugated by a flexible interdomain linker that provides a certain degree of movement to the active configuration of RNAP. It has been shown that the extension or deletion of the linker domain can alter RNAP affinity towards certain DNA promoters [31]. While this subunit mainly acts as the predecessor of RNAP assembly, the N- and C-terminal domains of the α subunit have different roles. The N-terminal domains from both α subunits form a dimer and act as a hydrophobic platform for subunit β and β′ binding [32,33]. The role of C-terminal domain is for molecular signalling between the enzyme with the class I transcription factor, and it also interacts with the AT-rich promoter upstream element that can provide an enhancement of RNAP activity [34,35].

The function of β and β′ subunits is for the claw-shaped core of RNAP. The β subunit has a 150 kDa size, which is slightly smaller than a 155 kDa β′ subunit. Both of these large proteins bind with the α subunits dimer in an organized manner, where the C-terminus of the β subunit is positioned near the N-terminus of the β′ subunit. Each of these subunits have a double-psi beta-barrel motif as a part of the RNAP active site; the active site coordinates with the Mg (II) ions utilizing the aspartic acid triad to facilitate the nucleotidyl transfer reaction [36]. Residues 47–172 and 375–428 are in the β1 domain, while the β2 domain contains residues 177–370; both of these domains together with the β′ clamp generate the DNA/RNA hybrid binding site. As the pivotal point of RNAP activity, the dynamics of this conformation are tightly controlled by CarD and RbpA as a part of the transcription factors. CarD binds to the β1 and β2 domain about ~70 Å away from the Mg^2+^-containing active site and acts as a tether between the RNAP and promoter DNA template. In contrast, RbpA binds to the other side of the β subunits using residues 478–677, contributing to the stability of the initiation complex [37,38]. Aside from providing the binding channel for the DNA promoter, the β subunit is essential in promoter recognition. For this purpose, the β-subunit flap domain at residues 855–914 and the β′ coiled-coil region at residues 262–309 interact with the sigma factor, promoting configurational transition. During this allosteric change, the σ2 domain is positioned close to the β′ coiled-coil region, while the β-flap domain interacts with the σ4 domain. This specific placement positioned between the σ2 and σ4 domains, which matches with the distance between the -10 and -35 non-template strand element, facilitates the specific promoter recognition [39,40].

MTB RNAP is a zinc metalloenzyme, and the zinc-binding domain (ZBD) possesses a prominent role in the transcription process. The ZBD is located inside the β′ subunit and is characterized by four cysteine residues that coordinate with the zinc ions. Another characteristic is that this domain has positively charged residues. The transcription factor RbpA binds to this domain, indicating its importance in transcription regulation. Other than the regulatory effect, this domain might interact directly with the DNA promoter within the spacer region to strengthen the interaction between the -35 element and the σ4 domain [41]. This is supported by the fact that the mutation study of *E. coli* β′-ZBD resulted in weaker promoter binding and lowered the ratio of stable initiation complex formation [42]. In agreement with this, the structural study also showed polar interaction between the β′-ZBD and promoter spacer region [43,44].

The catalysis of RNA synthesis involves transfer of the nucleotidyl from the NTP substrate that is bound to the active site using the 3′-OH ends of the newly synthesized RNA. After the transfer is complete, RNAP moves to accommodate the transfer of the next complementary nucleotide in the template sequence. The mobile domain of the β′ subunit regulates this cycle. This mobile domain can switch between the ‘trigger loop’ and ‘trigger helix’ conformations. The loop conformation allows NTP entry to the active state; after the change to the helix conformation, the channel is closed, and the transfer reaction starts. Other domains of the β′ subunit, called ‘fork loop’ and ‘bridge helix’, can make contact with the mobile domain to modulate coordination of the nucleotide addition cycle [45].

During the RNAP core enzyme assembly, the ω subunit is the last and smallest subunit added to the complex. The ω subunit remains as the least-studied subunit; it was first thought that this subunit is not necessary for the transcriptional activity. A knockout study of the *E. coli* ω-coding gene (*rpoZ*) once revealed that the core RNAP without ω subunit appeared to be morphologically the same [46]. It was known later that the ω subunit has a specific role in the assembly and activity of RNAP. RNAPs with the ω subunit were reported to recover quickly from harsh, denaturing conditions compared to those without [47]. The destabilization effect was also observed through the deletion study of the C-terminal region of the β′ subunit, the binding site for the ω subunit. Elimination of the ω subunit in the MTB core RNAP interrupted protein assembly and affected the activity of MTB RNAP [48]. This phenomenon was not seen in other bacteria such as *M. stegmatis* and *E. coli*, where the stabilization role of this subunit can be substituted with another factor such as *GroEL* homologs in *E. coli* [49,50]. It is hypothesized that the mycobacterial ω subunit might have a more important role compared to the ω subunit of other organisms.

Initially, studies on mycobacterial transcription were conducted based on an *E. coli* transcription model, due to the difficult handling of the mycobacterial pathogen. However, the usage of an *E. coli* model for antimycobacterial study was found to be ineffective since the structural shift might make a significant difference in the activity study. For example, RIF sensitivity towards MTB RNAP is 1000-fold higher compared to *E. coli* RNAP, although RIF was found to bind tightly to the same binding site in *E. coli* RNAP [19]. Structural studies for mycobacterial RNAP have emerged and several established models of mycobacterial RNAP are now available.

### 2.2. MTB Transcription Factors

Regulating the transcription process helps bacteria to defend themselves from the host’s defence mechanisms that prevent their growth during the infection. As one of the organisms with complex infection and adaptation mechanisms, MTB has distinct and highly coordinated regulatory features [51]. The transcription process in MTB is controlled by a number of regulatory factors, including sigma (σ) factors, RelMtb, RNAP-binding proteins (i.e., CarD, RbpA, Nus), essential Two Components Factor (TCF) and iron-binding Transcription Factor (TF), and some non-essential transcription factors [52]. In this section, the druggable transcription factors are discussed, including σ factor, CarD, RbpA, and Nus protein.

#### 2.2.1. Sigma Factor

Sigma (σ) factor proteins play a significant role in the MTB transcriptional process. These proteins bind reversibly to the RNAP core and form the holoenzyme during the initiation step. The specificity of RNAP is determined by the type of σ factor protein to which it is attached; various σ factor proteins direct RNAP to recognize a specific set of DNA promoters [53]. Based on the physiological roles, σ factors are categorized into primary housekeeping and accessory σ factors. The primary σ factor is typically involved in the expression of essential genes for growth, while the latter is mainly responsible for regulating a specific stress response [54,55,56].

Each type of bacteria expresses a different number of σ factor proteins, with most having a primary and multiple accessory σ factors [57]. MTB possesses 13 sigma factor genes, which encode for one primary σ factor (*sigA*), and 12 accessory of σ factors (*sigB*, *sigC*, *sigD*, *sigE*, *sigF*, *sigG*, *sigH*, *sigI*, *sigJ*, *sigK*, *sigL*, and *sigM*), which are associated with different tasks in the same signalling network [58]. *SigA* serves as the principal σ factor, and its role is for the transcription regulation of housekeeping genes. Moreover, *sigA* was found to be overexpressed in in vivo TB-infected pulmonary macrophage environments, indicating its role in regulating virulence-related genes during the early phase of infection [59]. *SigB* is closely related to *sigA* (62% homologous) and is believed to regulate more generalized stress conditions. A recent study indicated that *sigB* can recognize some housekeeping genes’ promoter regions during the exponential phase. This serves as evidence that *sigB* can act as a counterpart to *sigA* during this phase [60]. *SigF* was first thought to be upregulated in the case of nutrient starvation; however, global gene expression profiling revealed that *sigF* regulates the transcription of cell wall-associated genes such as MmpL, PE, and PPE families for survival during the host–pathogen interactions [61,62]. The remaining σ factors are a part of the most diverse and heterogenous extra cytoplasmic function (ECF) of σ factors, and they are involved in regulating the response during stress conditions, such as nitrogen depletion, heat/cold shock, malnutrition, hypoxia, and oxidative stresses [61,63]. During the lag phase in the pathogen’s reactivation, it is found that *sigE* and *sigH* are downregulated, indicating their importance in maintaining the MTB persistence in the non-replicating/slow-replicating state [64]. Relative to other pathogens, MTBs have a fairly high accessory sigma factor coding gene/genome size ratio, making them more adaptive towards a diverse environmental condition during infection [61].

Despite the disparity in function, the σ factors work in a collective manner, forming an intricate and multi-layer signalling network to regulate the RNAP. An integrated study to reconstruct this regulatory network revealed that there is a high connectivity between the components that are built through 41 direct interactions [65]. These interactions enabled MTB to exhibit specialized transcription regulation tailored to multiple growth phases, which makes this network an interesting inhibition target for antitubercular drugs. A previous attempt to inhibit this network has been demonstrated with the drug-repurposing study using Thioridazine, a dopamine receptor inhibitor drug. Thioridazine was found to have antitubercular activity against nutrient-depleted MTB, and it disturbed the σH/σE/σB network, which resulted in damage to the cell envelope [66].

#### 2.2.2. CarD

Some RNAP-binding proteins are known to regulate the MTB transcription process, such as CarD, RbpA, and NusG. CarD is conserved and essential in mycobacteria as a global transcription regulator. Structural analysis revealed that CarD stabilizes the open promoter complex formed between the holoenzyme and σA during the initiation step [67,68]. In this case, CarD has a role as a ‘bridge’ between promoter DNA and the RNAP. The N-terminal subdomain of CarD binds to the RNAP β subunit at the lobes, while the C-terminal subdomain recognizes the rRNA rrnAP3 promoter [69,70,71]. Within the C-terminus binding site, the bulky, hydrophobic nature of the W86 side chain facilitates Trp/thymine binding between the CarD and DNA promoter. A substitution study showed that replacing Trp with other hydrophobic side chains resulted in reduced or loss of activity for this protein. In good agreement with this fact, the W86 residue of CarD is conserved in more than 95% of CarD, indicating the importance of this residue [72].

CarD presence is essential in MTB, although this protein is interestingly absent in *E. coli*. *E. coli* and MTB share a similar transcription system, but the stability of their open promoter complexes is distinctly different. A single round abortive initiation assay for *E. coli* and MTB transcription revealed that mycobacterium open promoter complex decayed rapidly in the absence of CarD, unlike in *E. coli*, which still showed high transcription activity [70].

Inhibition of CarD/RNAP β subunit interaction was reported to influence the viability and susceptibility of the pathogen towards various antitubercular agents. A higher response to RIF was observed when the CarD/RNAP interaction was targeted, which encouraged the synergistic treatment for TB [73]. Although the mutations in the β subunit have allosteric effects on RIF binding in the case of DR-TB treatment, CarD/RNAP interactions still have great potential as antitubercular drug targets. Other than the stabilization of the open promoter complex, the alteration of CarD activity also exhibited a major divergence in gene expression of the MTB genome, leading to the hypothesis that CarD may regulate critical homeostasis in MTB [71]. Several attempts have been made to search for inhibitors of CarD/RNAP interaction. One recent study utilized a high throughput screening assay with labelled CarD to quantify its association with RNAP and promoter DNA using a fluorescence polarization (FP) assay [74]. Another effort was recently made to design a peptide-based inhibitor to inhibit this protein–protein interaction [75].

#### 2.2.3. RbpA

As another transcription factor, RNAP binding protein A (RbpA) is firstly found to be specific to Actinomycetes. Like CarD, RbpA is essential for MTB growth, but it is absent in *E. coli* [76]. MTB RbpA has a dimeric structure with a relatively small size and comprises 111 amino acid residues. It consists of an N-terminal tail domain (NTT), core domain (CD), basic linker (BL), and sigma interaction domain (SID). In M. stegmatis, RbpA influences RIF affinity towards the RNAP binding site and increases the resistance level due to the proximity of *M. stegmatis* and RbpA binding site (residue R381 on the β subunit) to the RIF binding site on the β subunit [77,78]. In MTB, it was found that RbpA did not directly influence RIF tolerance. The MTB RbpA binding site is found to be near the Sandwich-Barrel Hybrid Motif, which is distant from the RIF binding site. Moreover, the calculated IC_50_ of RIF from transcription initiation assay with and without the presence of RbpA shows constant inhibition (IC_50_: ~10 nM) [37]. Structural analysis showed that RbpA can selectively bind to the σ and β subunit of MTB RNAP. RbpA is also able to interact directly with the promoter DNA through van der Waals and electrostatic interactions with the BL domain. It was later proven that DNA–RbpA interaction is prominent for the transcription initiation from the loss of activity in the assay with MTB RbpA-R79A mutant [79].

**Figure 2:**
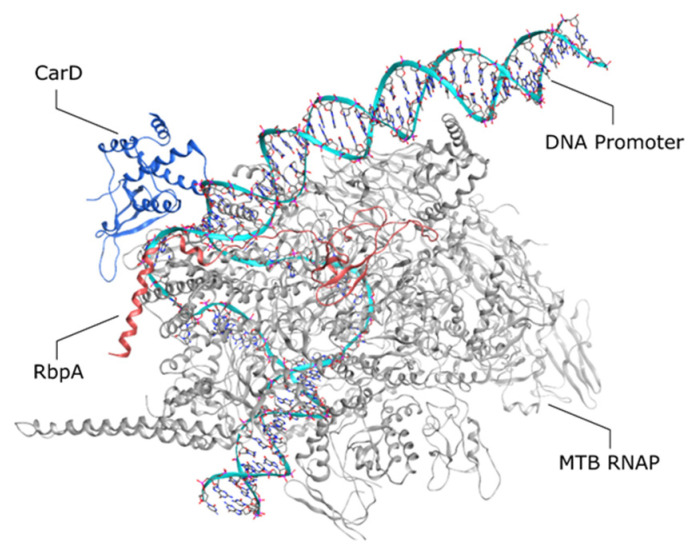
CarD, RbpA/RNAP binding interaction, MTB RNAP open promoter complex with AP3 promoter (PDB ID: 6EDT [82]). AP3 promoter strand: light blue, CarD: solid blue ribbon, RbpA: red ribbon.

During the initiation step, it was found that RbpA synergistically works with CarD to stabilize the open promoter complex formation through a different mechanism of action. Both RbpA and CarD bind to a different binding site on the complex (Figure 2), which works on the opposite sides of the DNA, with a different kinetic characteristic. Although RbpA exhibited lower affinity towards the complexes and did not show any stabilization effect while tested alone, a combination of CarD and RbpA extends the half-life of the open promoter complex two times higher than that of CarD alone [80,81].

In opposition to the BL and SID, other domains of RbpA such as NTT and CD were found to act antagonistically towards the open promoter complex stabilization. The deletion of both domains was proven to increase the complex’s stability, but it is believed that NTT and CD could contribute to RNAP activity through a distinct mechanism due to the strategic position they hold on the RNAP-σA complex. A recent study showed that RbpA NTT influences Fidaxomicin (Fdx) activity towards RNAP. Fdx inhibits MTB RNAP by blocking the clamp movement and holds it in the open clamp formation, preventing the initiation step. It was revealed that the E17 and R10 residues on RbpA NTT provide additional contact with Fdx, resulted in stronger interaction on the Fdx binding site [83]. This result offered some insights, such as the impact of retaining a low stability for the closed clamp formation on the RNAP complex. This, along with other regulation mechanisms that allow to keep the clamp open for longer; can be another strategy to improve Fdx activity or to develop another antitubercular compound.

#### 2.2.4. NusG

After the initiation process is completed, the σ factor detaches from the initiation complex to form the elongation complex. During elongation, RNAP moves along the DNA template to synthesize the new RNA. This dynamic is regulated by some elongation factors, one being the group of N-utilization substances (Nus) protein. Four proteins belong to this class, including NusA, NusB, NusE, and NusG. This class is known to promote antitermination processes in transcription and remains as a potential target for antibiotics. The Nus proteins are potential targets because they bind to the elongation complex and remain there until the elongation is completed. Aside from playing a part in anti-termination, they are capable of regulating the elongation pause and termination processes [84,85,86,87]. Among the Nus proteins, NusG is a highly conserved transcription factor throughout all species and has been found to directly influence the elongation rate. NusG consists of N- and C-terminus domains conjugated via a flexible linker [88]. A study on *E. coli* NusG revealed that this protein can interact with RNAP at the central cleft, with the N-terminal domain bound at the upstream fork junction. This position ensures clamp closure and is hypothesized to promote the elongation complex stabilization [89]. Targeting NusG/RNAP interaction might also interfere with the sigma–RNAP interaction and lead to the disruption of the stimulation of intrinsic termination regulated by this protein [16,90].

## 3. MTB Transcription Mechanism

Bacterial transcription is a multi-step process that consists of initiation, elongation, and termination. These processes are initiated by the formation of the RNAP holoenzyme, where the core enzyme (α2ββ′ω) binds to the σ factor protein. Afterwards, the initiation step takes place, where the RNAP holoenzyme recognizes a specific DNA promoter sequence and triggers the DNA strand to unwind. After the template DNA is exposed, nucleotide addition occurs during the elongation phase producing the nascent RNA strand. This process is terminated (termination step) once the enzyme recognizes a termination sequence in the template, releasing the newly synthesized RNA product. In this section, the dynamics and kinetics characterization of MTB RNAP during the transcription process are discussed.

### 3.1. Initiation

**Figure 3:**
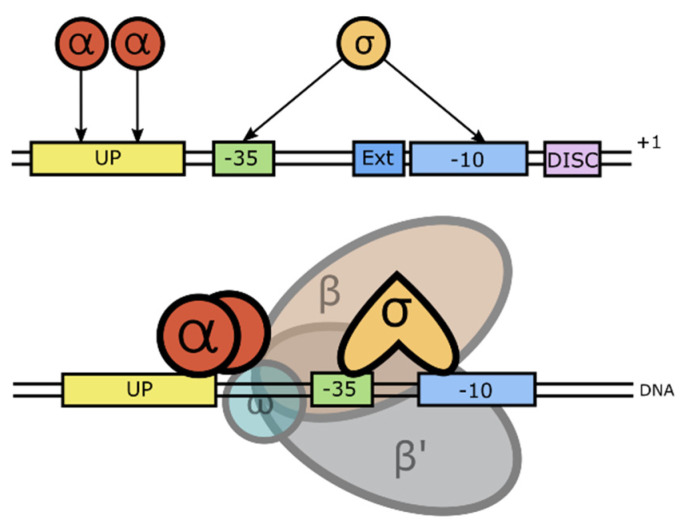
Schematic diagram of promoter recognition by the σ factor and the α subunit.

Binding of the sigma factor protein to the RNAP enables this enzyme to form specific binding with the promoter DNA and initiates the transcription process. During the initiation, MTB RNAP undergoes a number of conformational changes to form a closed promoter complex (RPc). This further stabilizes into an open promoter complex (RPo) to finally generate the initiating complex (RPitc). RPc formation is induced when the promoter DNA is attached to the RNAP. In this step, the σ factor protein and the α subunit of RNAP both play important roles in holding the promoter DNA together close to the RNAP β/β′ cleft to facilitate the transcription bubble formation in the next step [56]. The σ protein is known to recognize the -10 and -35 element sequences, as well as the TG sequence located upstream of -10 of the promoter DNA sequence [57,91]. Meanwhile, the α subunit interacts with the UP element where the A/T-rich sequence is located upstream of the -35 element—at its flexible C-terminal domain [92]. The promoter recognitions by these two subunits are visualized in Figure 3.

The RPc formation induces a series of changes to generate the catalytically competent complex RPo. These changes aim to unwind the dsDNA strand and place the template strand on the active site of RNAP to initiate the RNA synthesis. A recent study revealed the mechanism of DNA unwinding during the RPo formation by observing a single-molecule fluorescence resonance towards *E. coli* RNAP during the transcription initiation [93]. From this result, it is believed that after the promoter binds, the RNAP clamp remains closed during the unwinding and loading of the single-stranded template DNA to RNAP cleft. The promoter DNA unwinds in an upstream-to-downstream manner outside the active site, and after the ssDNA is fully loaded into the active site, the clamp closure is tightened to trap the ssDNA inside. The transcription bubble contained an opened dsDNA strand of about 12–13 base pairs. Structurally, during this unwinding and loading process, the interaction between sigma factor with the -10 element of the promoter DNA detached and shifted to the non-template strand from -11 to -7. Other domains of the σ factor also interact with position -6 and -5 on the non-template strand after the transcription bubble formation. Sigma factor protein does not interact with the template strand and influences the promoter escape in the elongation step [94].

The MTB RPo is known to dissociate rather fast, after ~1 to 2 min; the stabilization is needed to maintain an effective transcription rate. As mentioned before, transcription factors CarD and RbpA have been revealed to work together to restrict the movement of RNAP/promoter interactions and avoid the collapsing of the transcription bubble. During RPo formation, RPc undergoes isomerization, forming two intermediates before it forms the stable RPo. CarD and RbpA regulate this process by increasing the second intermediate formation rate, bypassing the energy barrier to form more stable RPo. This is different from other transcription activators such as CRP that play a part during the initial promoter-recognition step [70,95,96,97]

**Figure 4:**
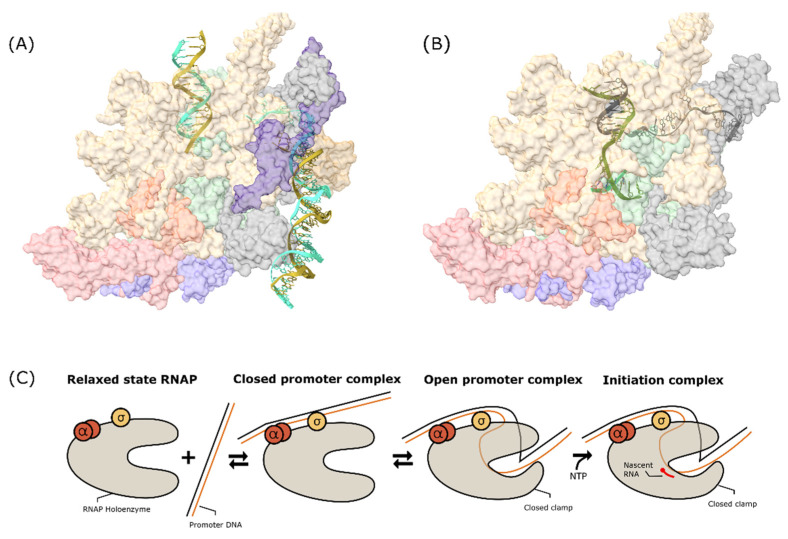
(**A**) MTB RNAP open promoter complex with WhiB7 promoter (PDBID: 7KIN [98]). (**B**) MTB RNAP initiation complex with 4nt RNA (PDBID: 5UH8 [99]). The green ribbon shows the growing RNA transcript inside the active site. (**C**) Schematic diagram of conformational change of RNAP during transcription initiation.

Transcription bubble formation in the RPo triggers RNA synthesis from the template DNA. This process generates a covalent phosphodiester bond between the 3′OH end of the newly synthesized RNA and the α-phosphate on the added nucleotide to increase one nucleotide to the chain, followed by the release of pyrophosphate as a side product. At the beginning, interaction between RNAP and promoter is still maintained. More of the template strand is dragged inside the transcription bubble, resulting in the expansion of the strand and the addition of tension to the ‘scrunched’ promoter DNA. As a result, the promoter DNA is detached from the initiation complex to release the tension, and the σ factor is also known to be dissociated from the complex, triggering the formation of an elongation complex. Figure 4 shows the conformational changes during initiation from the relaxed state to initiation complex formation.

### 3.2. Elongation

**Figure 5:**
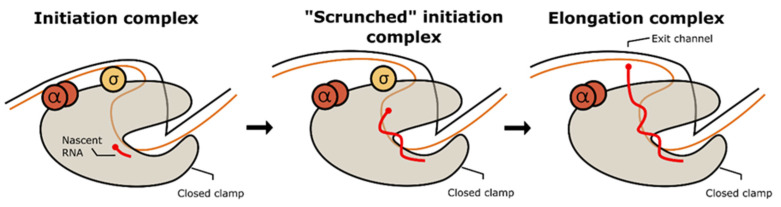
Schematic diagram of promoter escape and elongation complex formation.

Elongation involves continuous DNA duplex melting and the addition of subsequent base pairs to the opened strand. A productive and undisrupted elongation step ensures that the pathogen synthesizes the full-length sequence to make a functional protein, which supports the pathogen’s living functions. In the case of non-abortive initiation, the promoter escape and the σ factor dissociation act as the hallway for the stable ternary elongation complex formation. During the elongation, MTB RNAP moves through the single-stranded template in the 3′ to 5′ direction, adding one complementary nucleotide from the secondary channel each to the long nascent RNA product. The new RNA transcript is unloaded from the complex through a narrow hole formed by the β-flap domain and zinc-binding domain. This movement should be energetically balanced, as well as highly coordinated with the incoming NTP substrate. In MTB RNAP, auxiliary transcription factors such as the Nus protein control this process to avoid elongation failure [86,100,101]. The conformational change during the promoter escape on the ‘scrunched’ initiation complex and elongation complex formation is illustrated in Figure 5.

### 3.3. Termination

The elongation complex continues to move through the template until it recognizes a termination sequence. Bacteria have two types of transcription termination: intrinsic termination and factor-dependent termination. Intrinsic termination occurs due to the instability of the ternary elongation complex caused by the weak RNA/DNA hybrid, which is called ‘U-tract’. ‘U-tract’ is a 7–9 nucleotide long sequence that is composed by a GC-rich hairpin, followed by uracil-rich tract. This specific sequence results in the synthesis of the stem-loop structure and induces transcript release by the TEC to terminate the transcription process. Another mechanism involves the Rho termination factor, an ATP-dependent RNA helicase. Rho factor binds to the Rho utilization (rut) site on the newly synthesized RNA, moving through the RNA chain and dissociating the TEC [102,103].

## 4. Drug Development against MTB Transcription

Understanding the mechanism, structural characteristics, and conformational dynamics of the transcription process provides the insights needed to develop a potential inhibitor for MTB. In this section, several compounds that have been known to inhibit bacterial RNAP are summarized in Table 1, according to the step that they targeted.

Fidaxomicin, also known as Lipiarmycin A3, Tiacumicin B, or OPT-80, is a glycosylated macrocyclic lactone compound that was approved for treating gastrointestinal infection caused by Clostridium difficile [124]. It is known to bind to the conserved ‘switch region’ of RNAP, which is positioned at the base of the RNAP clamp. The conformational change within this region is associated with RNAP clamp dynamics, which affect DNA promoter loading to the RNAP active site. Upon binding, Fidaxomicin disrupts the conformational changes by ‘jamming’ the RNAP open clamp conformation. Due to this mode of action, Fidaxomicin inhibits RNAP in the early stage of transcription and does not inhibit the later stage (when the clamp is in a closed conformation) [125,126].

The potency of Fidaxomicin as an antitubercular agent was initially characterized during a natural product screening for antitubercular agent, where this antibiotic was identified as a hit compound [127]. In an inhibitory activity study, it was found that this compound has better antimycobacterial activity compared to streptomycin, but similar to moxifloxacin. Fidaxomicin was also found to be active against clinical MDR-TB strains and did not exhibit any cross-resistance with the first-line drugs INH and RIF [30]. Despite its potent activity, the utilization of Fidaxomicin as a TB drug is limited by its poor solubility. Previous attempts to structurally modify fidaxomicin have been made to address this limitation by synthesizing fidaxomicin derivatives and further testing using fluorescence-based assay [128,129].

Myxopyronin and corallopyronin are α-pyrone antibiotics derived from myxobacteria [130,131]. Both of these compounds were found to be bactericidal against Gram-positive microorganisms (MIC against MTB RNAP < 12.5 µg/mL), exhibit no cross-resistance with RIF, and have no effect on eukaryotic RNAP [131]. The antibiotics belonging to this class are relatively easier to synthesize compared to the macrocyclic antitubercular natural product, such as RIF. Structural studies showed that these antibiotics bound to the switch region of RNAP, and it was firstly proposed that myxopyronin inhibits RNAP activity by preventing the clamp opening motion during the initiation. Through another structural study, it was also hypothesized that myxopyronin prevents the DNA template entry by stabilizing the refolding of the switch-2 segment of the β′ subunit of RNAP, resulting in incompatible configuration for DNA accommodation [107,132]. A cryo-EM structure of MTB RNAP in complex with corallopyronin solved in 2019 revealed that both proposed mechanisms might contribute to the mode of action of these compounds, as corallopyronin was found to close the MTB RNAP clamp upon binding and prevent the later step of promoter melting [82].

Several clinical challenges have been addressed regarding the usage of these α-pyrone compounds as antitubercular agents. The bactericidal activity was found to dramatically decline in the presence of serum albumin due to hydrophobic interaction. It was later studied that myxopyronin also interacts with RNAP, mainly through hydrophobic interaction, which eliminates the possibility of developing a less hydrophobic analogue of this compound [132,133]. Another issue is related to compound stability, in which myxopyronin was found to be unstable on a lower pH and under UV light exposure [133]. An attempt to develop a hybrid inhibitor of myxopyronin was made by Yakushiji et al. in 2013 by incorporating holomycin antibiotic scaffold to myxopyronin skeleton [134]. From 38 compounds, only one was identified to have a comparable activity toward *E. coli* RNAP.

Ripostatin is a polyketide-derived macrolide with no structural similarity to the α-pyrone RNAP inhibitor [135]. Ripostatin also inhibits bacterial RNAP through the switch region with a similar mechanism to myxopyronin and corallopyronin, which was proven by the high cross-resistance. Ripostatin was initially showed to be active against MTB RNAP in vitro, but when studied against MTB culture, it was proven otherwise. As it was thought that bacterial cell wall permeability might be the issue, a structural modification attempt was conducted to improve the activity [136]. From ripostatin analogue synthesis, the carboxylate group was identified to be non-essential for the bactericidal activity. The hydrophobic tail-truncated analogue of ripostatin was also found to be inactive. Incubation with efflux pump inhibitor was also attempted to see whether the inactivity was caused by the MTB efflux mechanism, but no activity was observed, suggesting that permeability might not be the sole reason for the lack of activity.

Sorangicin is a macrolide polyether natural product isolated from Sorangium cellulosum [110]. Sorangicin binds to the RIF binding site in bacterial RNAP, despite having no structural similarity to RIF. This compound was found to not inhibit eukaryote RNAP and works against MTB RNAP with the same inhibition mechanism as RIF by preventing the translocation of short RNA transcript (around 2–3 nucleotides), leading to abortive initiation. Interestingly, not all RIF-resistant strains are sorangicin-resistant. Sorangicin inhibits RIF-resistant RNAP through a different mechanism shown by the absence of abortive products formed during the transcription assay against S456L mutant RNAP [109]. Structural study revealed that sorangicin blocks the single-stranded DNA template to reach the catalytic centre of the RNAP mutant at an earlier step of initiation compared to that of RNAP WT. In terms of inhibitory activity, sorangicin was known to have a similar sensitivity compared to RIF (IC50 against MTB WT; RIF = 0.010 µM, sorangicin = 0.033 µM). Pharmacokinetic study showed that sorangicin is not a potent CYP3A4 inducer and exhibited lower potential for drug–drug interaction compared to RIF, rifabutin, and rifapentine. This result makes sorangicin an attractive compound for the next-generation TB drug, as RIF is known as a strong CYP inducer and has a broad effect in drug–drug interaction [137].

Streptolydigin is a tetramic acid antibiotic, which has been found to inhibit nucleotide addition in the initiation and elongation step of bacterial transcription [138]. The binding site of streptolydigin partially overlaps with the RIF binding site and exhibits no cross-resistance with sorangicin or microcin J25. It is hypothesized that streptolydigin interferes with the RNAP translocational state. Structural study showed that streptolydigin interacts with the bridge helix and trigger-loop region in bacterial RNAP, away from the magnesium-containing active centre. From the bacterial RNAP-streptolydigin complex, it was observed that the bridge helix adopted a straight conformation upon binding. The alternating conformation of the bridge helix domain (between straight and bent) is hypothesized to possibly influence RNAP translocation. It was firstly found to be active against MTB in culture using turbidity measurement, but despite the broad-spectrum activity of this compound, re-testing using Nitrate Reductase Assay (NRA) and Microplate Alamar Blue Assay (MABA) showed that streptolydigin and its derivatives were found to be inactive (MIC more than 10 mg/L) [112,139].

Prokaryotic Gre proteins are transcription factors that work by stimulating the endogenous cleavage of aberrant 3′ end transcript [140]. In MTB genome, Rv1080c was known to share high sequence similarity with *E. coli* Gre factor. An affinity pulldown assay and in vitro transcription assays confirmed the ability of this protein to bind with RNAP and the transcript cleavage activity. Another gene, Rv3788, was also found to share a lower degree of similarity with *E. coli* GreA compared to Rv1080c [141]. Rv3788 protein has a similar domain organization to the Gre factor but lacks the cleavage ability. Instead, Rv3788 inhibits the transcription of various MTB promoters during ternary complex formation. The inhibitions were specific to MTB, as this protein was found to be inactive towards *E. coli*. Structural study showed that Rv3788 inhibited transcription through its N-terminus domain that fit in the narrowest region of the secondary channel and blocked the nucleotide entry to the RNAP active site. The inhibitory activity of Rv3788 was significantly reduced in the presence of MTB Gre, showing that they competed for the same binding site in MTB RNAP [113].

Another compound known to bind to the secondary channel of MTB RNAP is D-AAP1 (N-α-aroyl-N-aryl-phenylalaninamides). This compound belongs to a novel class of RNAP inhibitor found from the high-throughput screening of synthetic compounds using promoter-dependent fluorescence-based transcription assay. D-AAP1 binds the bridge helix binding site deep inside the secondary channel, and conformational change in this site is essential to accommodate NTP uptake during the transcription [99]. While it has proven to be potent against WT- and RIF-resistant mutants, this small molecule is also mycobacteria selective (inactive against other bacterial and mammalian cells). The co-administration of RIF and D-AAP1 exhibited no cross-resistance and the simultaneous binding resulted in additive antimicrobial activity.

Rifamycin is an important class of antibiotics with a wide spectrum of activity. Rifamycin compounds are known to target bacterial RNAP with a well-established inhibitory mechanism (detail reviewed in [142]). RIF, the first line drug for TB, belongs to this category and binds to the RIF binding site adjacent to the RNAP active site, sterically blocking the growth of newly synthesized RNA transcript after 2-3 nucleotides. All clinically used semisynthetic RIF analogues (i.e., rifalazil, rifabutin, rifapentine, and rifaximin) also work with the same mechanism. These analogues were designed from structural modification focused on the naphthoquinone ring to improve the therapeutic behaviour of RIF [143].

Kanglemycin A is a natural rifamycin isolated from soil, and its antimicrobial activity was identified first through a disk diffusion assay. This compound has a distinct structural feature compared to the existing semisynthetic rifamycins, with modifications on the ansa chain instead of the naphthoquinone moiety. Kanglemycin A has 2,2-dimethyl-succinic acid substitution on the C20 position and a unique sugar β-O-3,4-O,O′-methylene digitoxose on the C27 [114,115]. These substitutions lead to a larger binding surface and different mechanism of action than RIF, although they bind to the same binding site. Unlike RIF, in vitro transcription assay for kanglemycin A did not show any trace of 2-3 nucleotides abortive product. Mechanistic study showed that the bulky C20 side chain of kanglemycin A occupies the placement site of the initiating RNAP, inhibiting the initial dinucleotide synthesis. The sugar moiety on C27 reaches out to the unexplored hydrophobic pocket in the RIF binding site to improve the binding property.

Despite sharing the same binding site with RIF, kanglemycin A is active against RIF-resistant mutants of MTB RNAP. Structural study showed that the unique substitution on the ansa chain mediates the binding towards the mutant by forming new interactions with residues that do not interact with RIF. The sugar side chain interacts with residues R173 and T433 of MTB RpoB, and the succinic acid side chain forms a salt bridge with R604. Derivatization to the succinic acid chain has been performed in an effort to improve the bioavailability and in vivo efficacy of this compound, which resulted in better potency against WT bacteria, but loss of activity against the RIF-resistant mutants [116].

GE23077 is a naturally occurring cyclic heptapeptide, isolated from Actinomadura sp. [144]. This compound shows a potent nanomolar activity against *E. coli* RNAP and exhibits a narrow spectrum activity towards other bacteria, possibly because of poor penetration [118]. In vitro assay showed that GE23077 acts at the level of transcription initiation. Further study revealed that this peptide occupied a binding site that overlapped with the Mg-containing RNAP active site and the i + 1 nucleotide binding site [119]. This site is unoccupied in the open promoter complex but occupied in the elongation complex. To address the permeability, structural optimization was performed to three unnatural amino acids in GE23077 to change the total charge and increase the lipophilicity [145]. Alteration to α-amino malonic acid and β,γ-dihydroxyglutamine resulted in lower IC_50_ value, while alpha, β -diaminopropanoic acid (Apa) was found to be not critical for binding, as the GE23077 binding site is adjacent to the RIF binding site. The bipartite inhibitor GE23077-RIF was evaluated in the previous study, and the hybrid inhibitor was found to be more potent compared to GE23077 and RIF alone [119].

Microcin J25 is a ‘lasso’ peptide antibiotic that showed wide-range activity towards the *E. coli* transcription [146,147]. This peptide works by inhibiting the open promoter complex, initiation complex, and elongation complex. This compound binds to the secondary channel and prevents NTP uptake to the active site, disrupting the initiation complex formation. This compound also exhibited an inhibitory activity to the stalled elongation complex, indicating the ability to disrupt the elongation process [148,149]. Microcin J25 is stable against heat and harsh pH and is known to be resistant to many proteases [150]. A systematic mutational study was conducted to identify the structure–activity relationship, which revealed that not all amino acids in the sequence were strictly essential for the activity [151]. Gly1 and Glu8 are necessary for the lactam ring formation to conserve the ‘lasso’ conformation, while Gly2, Glu4, and Pro7 are essential for bacterial growth inhibition. Another mutational study to randomize the amino acid on the Ala3, His5, Val6, Gly12, Ile13, and Thr15 position confirmed that Microcin J25 still retained the lasso structure after modification [152,153].

From Table 1, it is observed that more inhibitors were known to target the initiation step, where some of the compounds were also capable of disrupting the elongation step. It is plausible that the disruption of other factors could be a beneficial key to combat this disease and address the problem of the emerging drug resistance caused by natural adaptations against the first-line drug RIF. It is also worth mentioning that one compound could possibly block the transcription process with a different mode of action towards the different mutants, while interacting to the same binding site on the protein. Extensive study is needed to characterize the exact mode of action and possibilities of cross-resistance between the RNAP-inhibitor drugs. In the case of MTB with distinct membrane characteristics and complex efflux mechanisms, it is not unexpected for a broad-spectrum RNAP inhibitor to not work against MTB culture, as shown by the ripostatin, streptolydigin, and CBR. Instead, lead optimization could address this problem through structural modification or to develop a novel compound with the same scaffold but with a distinct permeability profile.

## 5. Conclusions

The transcription process is complex and essential in mycobacteria. This process is highly regulated to ensure a successive and accurate transcript production is translated into a functional protein for this pathogen’s survival. The multi-subunit MTB RNAP acts as the machinery for this process, bearing the transcription site at its active site. MTB RNAP undergoes several conformational changes during the initiation, elongation, and termination processes. These changes involve attachment or detachment to some transcription factors to coordinate the DNA-promoter melting and insertion to the binding site to start the RNA synthesis. Some compounds have been found to inhibit this complex process at a certain point, while an understanding of the mechanisms revealed the gaps in the development of a novel compound targeting a different step. Most RNAP inhibitors are known to target the initiation process. While proven effective, targeting PPI between the subunit and transcription factor or modulating the regulator function to delay promoter escape/create more stalled complex might also increase the abortive transcription rate. Other gaps may exist in the elongation complex, where the induction of pausing or backtracking could lead to incomplete transcription or missense mutation. Disrupting the termination step through misrecognition of the termination factor rho also disturbs the accuracy of protein synthesis.

## Figures and Tables

**Table 1:** Known bacterial RNAP inhibitor.

Target State	Compound	Studied on	Binding Site	Mode of Action	Reference
Free RNAP	Fidaxomicin (macrocyclic antibiotic)	*Clostridium difficile*, *E. coli*, MTB	RNAP base clamp	Inhibit clamp closure	[104,105,106]
Closed promoter complex	Myxopyronin (α-pyrone antibiotic)	*C. difficile*, MTB, *S. aureus*, *E. coli*, *Enterococcus*, *Pseudomonas*, etc.	RNAP switch region	Inhibit RNAP interaction with promoter DNA, prevent formation of open promoter complex	[107,108]
Closed promoter complex	Corallopyronin (α-pyrone antibiotic)	*M. stegmatis*, *S aureus*, *S. simulans*, *B. substilis*, *B. cereus*	RNAP switch region		[107]
Closed promoter complex	Ripostatin (macrocyclic-lactone antibiotic)	*S. aureus*, *E. coli*, MTB (inactive in MTB)	RNAP switch region		[107]
Closed promoter complex	Sorangicin (macrocyclic-lactone antibiotic)	*T. aquaticus*, *M. stegmatis*, MTB	RIF binding site: ~12 Å away from the active centre	Inhibit DNA-unwinding process on MTB mutant S456L	[109,110]
Closed promoter complex	Streptolydigin (tetramic acid antibiotic)	*T. thermophilus*, MTB (Inactive towards MTB)	Bridge helix binding site: 20 Å away from active site, overlap the bridge helix and trigger loop	May stabilize intermediate during isomerization to open promoter complex, inhibit open promoter complex formation	[111,112]
Open promoter complex	Rv3788 (Gre homolog)	MTB, *M. stegmatis*, *E. coli* (Does not inhibit *E. coli* RNAP)	Near secondary channel	Inhibits formation of ternary complex	[113]
Open promoter complex	D-AAP1 (small molecule, aryl-aroyl-phenylalamide)	MTB	Bridge helix binding site: N-terminus on RNAP bridge helix	Prevent conformational change of bridge helix N-terminal hinge that is necessary for nucleotide addition	[99]
Open promoter complex	Kanglemycin (Ansamycin)	MTB, *M stegmatis*, *S aureus*, *L. monocytogenes*, *S. epidermidis*	RIF binding site: ~12 Å away from the active centre	Inhibits binding of the initiating NTP	[114,115,116]
Transcription initiation complex	Rifampicin (Ansamycin)	Known broad-spectrum bacterial RNAP inhibitor		Inhibit synthesis of short RNA product >2–3 nt by steric clashes	[117]
Transcription initiation complex	GE23077 (cyclic peptide antibiotic)	*E. coli*, *S. aureus*, MTB, *T. thermophilus*	NTP binding site, RNAP active centre. Slight overlap with PUM binding site.	Inhibit first nucleotide addition	[118,119]
Transcription initiation complex	Sorangicin (macrocyclic-lactone antibiotic)	*T. aquaticus*, *M. stegmatis*, MTB	RIF binding site: Near active centre, ~12 Å away from the active centre	Inhibit synthesis of short RNA product	[109,110]
Transcription initiation and elongation complex	Salinamide (depsipeptide antibiotic)	*S. aureus*, *E. coli*, *T. thermophilus*	Bridge helix binding site: N-terminal end of RNAP bridge helix at interface between RNAP β and β′ subunit	Prevent conformational change of bridge helix N-terminal hinge that is necessary for nucleotide addition	[120]
Transcription initiation and elongation complex	CBR703 (CBR hydroxamidine)	*E. coli*, MTB (Does not work in MTB or Gram-positive bacteria)			[121]
Transcription initiation and elongation complex	CBR Pyrazoles				
Open promoter complex, transcription initiation and elongation	Microcin J25/MccJ25 (cyclic peptide antibiotic)	*E. coli*	Secondary channel	Inhibit NTP binding on active site (NTP uptake, binding, phospodiester bond formation, or translocation)	[122,123]
